# Pathological Complete Response after Conversion Surgery for Distal Cholangiocarcinoma with High Tumor Mutational Burden and Para-Aortic Lymph Node Metastasis: A Case Report

**DOI:** 10.70352/scrj.cr.26-0221

**Published:** 2026-09-19

**Authors:** Ken Hatano, Yusuke Okamura, Kai Nakao, Gensho Tanke, Gen Yamamoto, Kenji Uryuhara, Masato Narita, Shigeo Hara, Satoshi Kaihara

**Affiliations:** 1 Department of Surgery, Kobe City Medical Center General Hospital, Kobe, Hyogo, Japan; 2 Department of Gastroenterology, Kobe City Medical Center General Hospital, Kobe, Hyogo, Japan; 3 Department of Pathology, Kobe City Medical Center General Hospital, Kobe, Hyogo, Japan

**Keywords:** distal cholangiocarcinoma, conversion surgery, tumor mutation burden high, durvalumab, pathological complete response, para-aortic lymph node metastasis

## Abstract

**INTRODUCTION:**

Pathological complete response (pCR) following conversion resection after chemotherapy for initially unresectable distal cholangiocarcinoma (DCC) is rare. The efficacy of the immune checkpoint inhibitors (ICIs) pembrolizumab and durvalumab for treating unresectable biliary tract cancer (BTC) has been reported in randomized trials, and they are used as first-line therapies, regardless of specific genetic mutations. High tumor mutation burden (TMB-High) has gained attention as a biomarker for predicting treatment response to ICI; however, it has been reported to be rare in BTC.

**CASE PRESENTATION:**

A 78-year-old man was referred to our hospital after mild liver dysfunction was detected during a routine medical checkup. Contrast-enhanced CT revealed wall thickening with contrast enhancement of the distal bile duct. Endoscopic ultrasonography revealed an irregular hypoechoic mass in the distal bile duct. Endoscopic retrograde cholangiopancreatography confirmed a distal bile duct stricture, and biopsy of the stricture site confirmed adenocarcinoma. Further evaluation revealed DCC with para-aortic lymph node (PALN) metastasis. The tumor was initially considered unresectable. Comprehensive cancer genomic profiling using tissue samples revealed TMB-High. The patient underwent 8 cycles of systemic chemotherapy consisting of gemcitabine, cisplatin, and durvalumab, followed by durvalumab monotherapy. After more than 1 year of chemotherapy, improvement in the bile duct stricture was confirmed, and complete disappearance of fluorodeoxyglucose uptake in the primary lesion and PALNs was observed. Based on these findings, conversion surgery was considered feasible, and subtotal stomach-preserving pancreaticoduodenectomy was performed. Histopathological examination revealed no residual tumor in the distal bile duct or PALNs. The therapeutic effect was grade 4, indicating pCR. The patient remains alive without recurrence 6 months after surgery.

**CONCLUSIONS:**

We encountered a case of TMB-High DCC in which conversion resection became feasible after systemic chemotherapy including the ICI durvalumab, resulting in pCR.

## Abbreviations

BTC
: biliary tract cancer
CA19-9
: carbohydrate antigen 19-9
CEA
: carcinoembryonic antigen
CECT
: contrast-enhanced CT
CGP
: cancer genomic profiling
CTLA-4
: Cytotoxic T-Lymphocyte-Associated Protein 4
DCC
: distal cholangiocarcinoma
EUS
: endoscopic ultrasonography
FDG
: fluorodeoxyglucose
GC
: gemcitabine/cisplatin
GCD
: gemcitabine/cisplatin plus durvalumab
GCP
: gemcitabine/cisplatin plus pembrolizumab
GCS
: gemcitabine/cisplatin plus S-1
ICC
: intrahepatic cholangiocarcinoma
ICI
: immune checkpoint inhibitor
PALN
: para-aortic lymph node
pCR
: pathological complete response
PD-1
: Programmed Cell Death Protein 1
PD-L1
: Programmed Death-Ligand 1
PFS
: progression-free survival
RR
: response rate
TMB
: tumor mutation burden
TMB-High
: high tumor mutation burden

## INTRODUCTION

BTC, including ICC, perihilar cholangiocarcinoma, DCC, and gallbladder cancer, are relatively rare malignant tumors; however, there are regional variations, and they are seen relatively more frequently in East Asian countries than in Western countries.^1)^ In addition, the incidence of ICC is increasing at an average annual rate of 2.3%, which is higher than the rate of increase for extrahepatic cholangiocarcinoma.^1)^ From the perspective of long-term survival, although surgical resection is the most promising treatment for BTC, the 5-year survival rate following resection is estimated to range from 11% to 44%.^2)^ Furthermore, less than one-third of patients are deemed resectable at the time of initial diagnosis, and many patients are diagnosed as inoperable due to distant metastases or locally advanced cancer.^3)^ Although chemotherapy has seen remarkable advances in recent years, the 2-year survival rate remains around 25%.^4–6)^

Remarkable progress has been made in the development of chemotherapy for unresectable BTC. The introduction of ICIs has also improved BTC outcomes. The TOPAZ-1 trial demonstrated a significant improvement in survival when durvalumab was added to GC therapy,^4)^ and the KEYNOTE-966 trial showed similar improvement with the addition of pembrolizumab.^5)^

Advances in CGP testing have enabled the measurement of TMB, which represents the total number of somatic mutations in the tumor genome.^7)^ High TMB (TMB-High) is gaining attention as a biomarker for predicting the therapeutic efficacy of ICIs; however, it is rarely seen in BTC, occurring in 0%–4% of cases.^8–10)^

Advances in chemotherapy and cancer genome analysis have increased the likelihood of performing conversion surgery after demonstrating the efficacy of chemotherapy, even in cases initially diagnosed as unresectable, suggesting the potential for improved prognosis.^11)^ However, cases of unresectable BTC that achieve pCR through conversion surgery are rare.

We report a case of DCC with TMB-High and PALN metastasis in which pCR was achieved following conversion surgery after GCD therapy.

## CASE PRESENTATION

A 78-year-old man was diagnosed with mild liver dysfunction during a routine checkup and was referred to our hospital for further evaluation. CECT revealed tumor and wall thickening with contrast enhancement in the distal bile duct, and dilation of the proximal bile duct (**Fig. 1A**). EUS revealed an irregular hypoechoic mass measuring 27 × 13 mm within the distal bile duct (**Fig. 1B**). Endoscopic retrograde cholangiography revealed a 3-cm stricture in the distal bile duct (**Fig. 1C**), and biopsy of the stricture site confirmed adenocarcinoma (**Fig. 2A**). A bile duct stent was placed for biliary decompression. CECT also revealed an enlarged PALN, which showed FDG uptake on PET (**Fig. 1E**) and high signal intensity on diffusion-weighted MRI, leading to a diagnosis of positive PALN metastasis. Based on these findings, the patient was diagnosed with unresectable DCC with PALN metastasis. The patient was Lewis antigen-negative and a non-secretor of CA19-9. The serum CEA level was 2.5 ng/ml, which was within the normal range (**Fig. 3**). The tumor resection was deemed ineligible, and GCD therapy was initiated as the first-line treatment. Comprehensive CGP analysis of the tissue sample using FoundationOne CDx 1 month after treatment initiation revealed TMB-High. As ICI therapy was deemed appropriate, the treatment regimen containing durvalumab was continued. After receiving 8 cycles of combination therapy, the patient was administered durvalumab monotherapy.^4)^ During treatment, the tumor-related bile duct stricture improved, allowing the removal of the previously placed biliary stent (**Fig. 1F**, **1H**). EUS revealed resolution of the wall thickening in the distal bile duct (**Fig. 1G**). After 9 cycles of durvalumab, PET-CT imaging demonstrated complete disappearance of FDG uptake in the primary cholangiocarcinoma and PALN metastases (**Fig. 1I**, **1J**). Conversion resection was planned (**Fig. 3**). Intraoperative frozen section analysis confirmed the absence of PALN metastasis, and radical resection via subtotal stomach-preserving pancreaticoduodenectomy was performed. The postoperative course was uneventful, and the patient was discharged on POD 13. Macroscopic examination of the horizontal section of the pancreatic head in the resected specimen revealed no wall thickening of the bile duct or tumorous lesions. Histopathological examination revealed no residual cancer in the distal bile duct. The therapeutic effect, as defined by the 7th edition of General Rules for Clinical and Pathological Studies on Cancer of the Biliary Tract in Japan, was grade 4, which was consistent with pCR (**Fig. 2**). Postoperative adjuvant chemotherapy was not administered. The patient remains alive without recurrence 6 months after surgery.

**Fig. 1:**
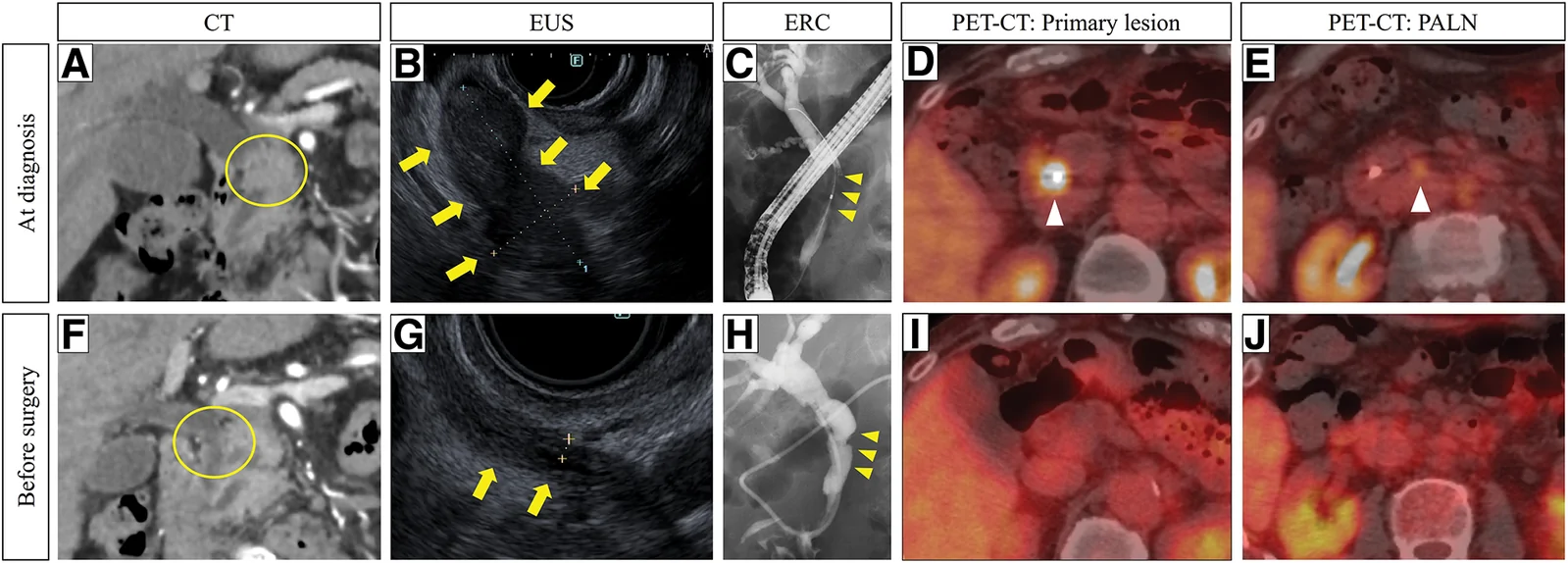
Imaging findings at diagnosis of distal bile duct cancer and prior to conversion resection (**A**–**E**, at diagnosis; **F**–**J**, before conversion surgery). (**A**) CECT revealed a mass (yellow circle) and wall thickening in the distal bile duct. (**B**) EUS revealed an irregular, hypoechoic mass measuring 27 × 13 mm within the distal bile duct (yellow arrows). (**C**) ERC demonstrated an approximately 30-mm stricture in the distal bile duct (yellow arrowheads). (**D**) PET/CT demonstrated abnormal FDG uptake in the distal bile duct tumor (white arrowhead). (**E**) Abnormal FDG uptake was also observed in the PALNs (white arrowhead). (**F**) CECT revealed a reduction in the size of the bile duct tumor (yellow circle). (**G**) EUS revealed resolution of the wall thickening in the distal bile duct (yellow arrows). (**H**) Cholangiography demonstrated resolution of the biliary stricture (yellow arrowheads). (**I**) PET/CT demonstrated resolution of abnormal FDG uptake in the primary lesion. (**J**) FDG uptake in the PALNs had completely disappeared. CECT, contrast-enhanced CT; ERC, endoscopic retrograde cholangiography; EUS, endoscopic ultrasonography; FDG, fluorodeoxyglucose; PALN, para-aortic lymph node

**Fig. 2:**
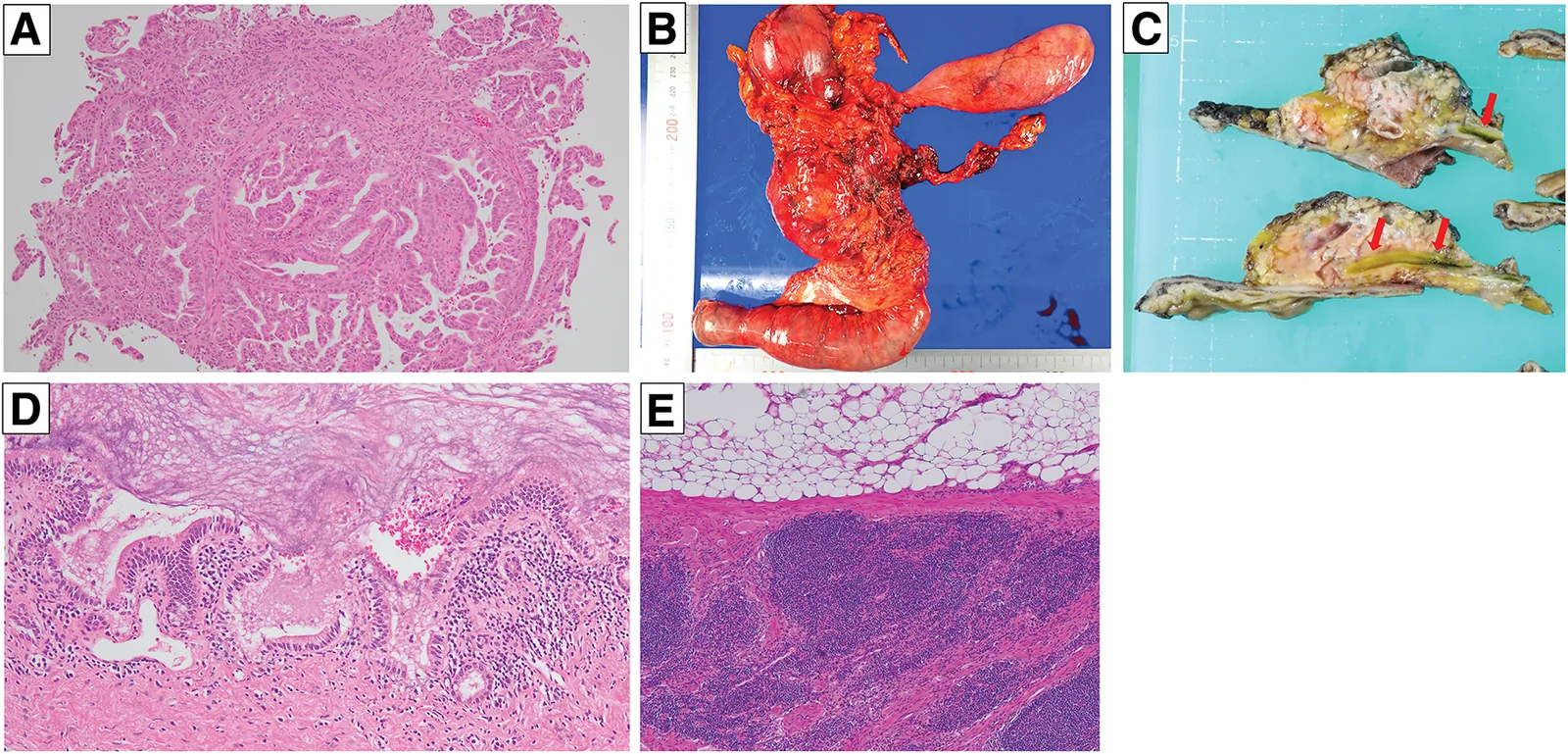
Histopathological findings at diagnosis and at conversion resection. (**A**) A biopsy specimen from the distal bile duct tumor site at diagnosis revealed atypical cells with enlarged nuclei forming glandular structures, consistent with adenocarcinoma (×100). (**B**) Macroscopic findings of a pancreaticoduodenectomy specimen. (**C**) Macroscopic findings on the horizontal section of the pancreatic head. The arrows indicate the distal bile duct, where no wall thickening or tumor was observed. (**D**) In the pancreaticoduodenectomy specimen, no evident tumor lesions were observed in the distal bile duct (×100). (**E**) No signs of malignancy were observed in the PALNs (×40). PALNs, para-aortic lymph nodes

**Fig. 3:**
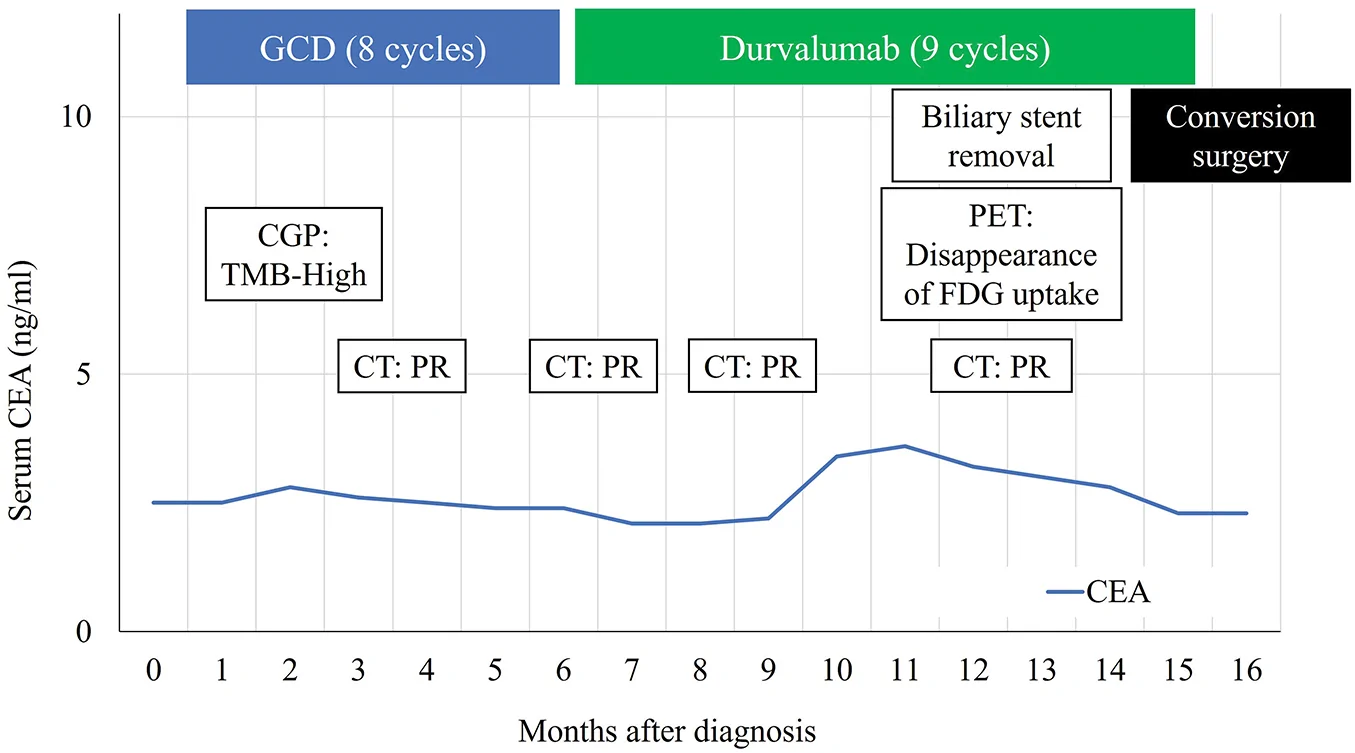
Clinical course from treatment initiation to conversion resection and changes in tumor markers. GCD therapy was initiated as the first-line treatment for unresectable DCC with PALN metastasis. Comprehensive CGP performed 1 month after treatment initiation revealed that the tumor was TMB-High. After receiving 8 cycles of combination therapy, the patient was started on durvalumab monotherapy. Three months after treatment initiation, a CT scan showed tumor shrinkage (PR), and the patient has remained in PR since then. After 9 cycles of durvalumab administration, the bile duct stricture improved, allowing for the removal of the bile duct stent. PET/CT scans confirmed the disappearance of FDG accumulation in the primary cholangiocarcinoma and the PALN metastases, demonstrating clinical CR. Subtotal stomach-preserving pancreaticoduodenectomy was performed using a conversion resection approach. Serum CEA levels remained within the normal range throughout the treatment period. CEA, carcinoembryonic antigen; CGP, cancer genomic profiling; DCC, distal cholangiocarcinoma; FDG, fluorodeoxyglucose; GCD, gemcitabine/cisplatin plus durvalumab; PALN, para-aortic lymph node; PR, partial response; TMB-High, high tumor mutation burden

## DISCUSSION

In recent years, conversion resection following chemotherapy for unresectable malignant tumors in the hepatobiliary–pancreatic region has become a topic of interest because of advances in chemotherapy. Favorable treatment outcomes have been reported for hepatocellular and pancreatic cancers.^12,13)^ However, there are few reports on BTC, and many aspects of the indications for and significance of conversion surgery remain unclear. This case is the first reported achievement of pCR in both the primary tumor and distant metastatic lesions following conversion surgery after GCD chemotherapy in a patient with TMB-High DCC that was initially deemed unresectable because of positive PALN metastasis. PALNs are defined as extra-regional lymph nodes in the Union for International Cancer Control (UICC), American Joint Committee on Cancer (AJCC) and Japanese classification of BTCs; a positive status corresponds to distant metastasis. Cases positive for PALNs alone are uncommon and typically involve regional lymph node metastasis, resulting in a poor prognosis.^14,15)^

The criteria for conversion surgery include tumor and patient factors. Among tumor factors, antitumor effects, such as tumor shrinkage or decreased tumor marker levels following nonsurgical treatment, are crucial. For BTC, no new potent treatment regimens have emerged since the ABC-02 trial, reported in 2010, established GC therapy as the standard treatment.^16)^ However, in recent years, 3 treatment regimens have been reported to be superior to GC therapy. The TOPAZ-1 trial, reported in 2022, demonstrated a RR of 26.7% and PFS of 7.2 months for GCD therapy (vs. 5.7 months in the GC group, hazard ratio [HR] 0.75, p = 0.001).^4)^ The KEYNOTE-966 trial, reported in 2023, demonstrated an RR of 29% and a PFS of 6.5 months for GCP therapy (GC group: 5.6 months, HR 0.86, p = 0.023)^5)^; and the KHBO1401-MITSUBA trial, reported in 2023, demonstrated an RR of 41.5% and a PFS of 7.4 months for GCS therapy (GC group: 5.5 months, HR 0.75, p = 0.015).^6)^ These results suggest an increase in the number of conversion resection cases. Maintaining antitumor effects over a certain period is also important. For pancreatic cancer conversion resection, a preoperative treatment period of 8 months or longer has been reported to be associated with a favorable prognosis.^17,18)^ Shorter control periods may also increase the risk of recurrence.

The implementation of personalized therapy based on CGP may increase the likelihood of conversion resection. In this case, TMB-High was confirmed by CGP, suggesting an association with the excellent antitumor effects of ICIs.^19)^ Although the efficacy of ICIs targeting CTLA-4, PD-1, and PD-L1 has been confirmed in advanced solid cancers with TMB-High,^10,20)^ TMB-High is rare in BTC, occurring at a rate of 0%–4%.^9,10)^ Durvalumab and pembrolizumab can be used as first-line therapies for all patients with unresectable BTC, regardless of specific genetic mutations. However, the importance of early genomic profiling during treatment has also been confirmed. **Table 1** summarizes 9 cases of pCR after ICI therapy for BTC. Among the 7 cases in which CGP was performed, significant findings were noted in 6.

**Table 1:** Nine Cases of UR-BTC Achieving pCR following Conversion Resection after ICI Therapy

Author	Year	Age	Sex	BTC	UR factor	Genetic mutation	First-line CTx	Second-line CTx	Recurrence	Survival (months)	Adjuvant CTx
Abundalou	2021	47	M	ICC	PALN	TMB-H	GCP	Pembrolizumab	No	>6	Pembrolizumab
Robinson	2024	49	M	ICC	Adrenal grand	TMB-H	GCP	–	No	>38	Unknown
Orlandi	2024	62	M	GBC	Local	None	GCD	–	No	>6	Unknown
Yasui	2025	70	M	PHCC	PALN	Unknown	GCD	–	No	>10	Unknown
Fukuda	2025	64	M	ICC	PALN	Lynch	GC	GCD	No	>10	No administration
Inokawa	2025	69	M	PHCC	Local	MSI-H	GC	Pembrolizumab	No	>4	No administration
Tomino	2025	54	F	PHCC	Local	Unknown	GCS	GCD	No	>4	S-1
Shirakawa	2026	50	F	ICC	Local	MSI-H	GCS	Pembrolizumab	No	>56	No administration
Present case	2026	78	M	DCC	PALN	TMB-H	GCD		No	>6	No administration

BTC, biliary tract cancer; CTx, chemotherapy; DCC, distal cholangiocarcinoma; F, female; GBC, gallbladder cancer; GC, gemcitabine/cisplatin; GCD, gemcitabine/cisplatin plus durvalumab; GCP, gemcitabine/cisplatin plus pembrolizumab; ICC, intrahepatic cholangiocarcinoma; ICI, immune checkpoint inhibitor; M, male; MSI-H, microsatellite instability high; PALN, para-aortic lymph node; pCR, pathological complete response; PHCC, perihilar cholangiocarcinoma; TMB-H, high tumor mutation burden; UR, unresectable

Regarding patient factors, maintaining systemic conditions, such as performance status and nutritional status, after chemotherapy is crucial.^17,21)^ In this case, tumor shrinkage was confirmed as an improvement in the bile duct stricture due to primary tumor reduction. PALN metastasis became negative, and tumor control was achieved after 15 months. As systemic conditions were maintained, the patient was deemed eligible for surgery. Previous reports have indicated a conversion resection rate of 4.9% in the GCS therapy cohort of the KHBO1401-MITSUBA trial^22)^ and 2.7% in a cohort of 475 patients receiving PD-1/PD-L1 antibody plus chemotherapy.^23)^

Several retrospective cohort studies have reported favorable outcomes of conversion resection for BTC. An international multicenter study of 56 cases of conversion resection for unresectable locally advanced BTC demonstrated a relatively favorable curative resection rate of 76.8% and a 5-year survival rate of 39.1%.^24)^ In the aforementioned study, cases exhibiting positive lymph nodes or distant metastases were associated with a poor prognosis. A systematic review reported that the conversion resection group exhibited a superior prognosis compared to the non-resection group.^11)^ In addition, with respect to conversion resection subsequent to PD-1/PD-L1 antibody-combined chemotherapy, as evidenced in the present case, it has been documented that when specific resection criteria were fulfilled, the resection group exhibited a more favorable prognosis than the group that continued chemotherapy. The effectiveness of chemotherapy is limited, and the significance of resection in cases amenable to conversion has been emphasized.^23)^ However, radical resection for BTC is a highly invasive procedure, with postoperative complication rates of grade 3 or higher at 60.7% and grade 3b or higher at 25%, and a mortality rate of 8.9%.^22,24)^ Consequently, indications should be meticulously evaluated on a case-by-case basis. The option of not performing conversion resection should also be supported.^25)^

Information regarding pCR rates in BTC is limited. In each pivotal trial, the complete RR was 2.1% for GCD,^4)^ 2% for GCP,^5)^ and 3% for GCS therapy.^6)^ In hepatocellular carcinoma, a correlation between pathological response and prognosis has been demonstrated in conversion resection cases, highlighting the importance of pCR.^26)^ **Table 1** summarizes 9 cases of pCR after ICI therapy for BTC. Although the significance of pCR in conversion resection cases for BTC remains unclear, all 9 cases shown in **Table 1** remained recurrence-free. There has been a case report of recurrence after surgery in a patient who achieved pCR only in the primary tumor but had lymph node metastases^27)^; therefore, it may be important to achieve a pCR not only in the primary tumor but also in the metastatic lesions.

The significance of postoperative adjuvant chemotherapy in cases of resected BTC that achieved pCR remains unclear. In the ASCOT trial,^28)^ which demonstrated the efficacy of S-1 as adjuvant chemotherapy for resected BTC, the study population consisted of patients with T2–4, N0, M0, or T1–4, N1, M0 (for patients with extrahepatic cholangiocarcinoma, gallbladder cancer, or ampullary cancer), or T1–4, N0–1, M0 (for patients with ICC). This trial did not include cases of pT0 or pTis. In the present case, since the duration of preoperative chemotherapy was approximately 1 year and was considered sufficient, adjuvant chemotherapy was not administered. Previous reports also indicate that the administration of adjuvant chemotherapy varies; among the 5 cases for which details were available, only 2 received it (**Table 1**). In cases of pCR, there is a possibility that chemotherapy can be discontinued, which may lead to a reduction in the risk of chemotherapy-related adverse events and lower treatment costs.

## CONCLUSIONS

Here, we reported a valuable case of unresectable TMB-High DCC. The patient underwent conversion resection after GCD therapy and achieved pCR. The possibility of conversion resection should always be explored in patients in whom the efficacy of durvalumab combination chemotherapy has been confirmed. Furthermore, early implementation of CGP is essential. Further accumulation of cases is expected to clarify the significance of performing conversion resection after ICI therapy.

## DECLARATIONS

### Availability of data and materials

The dataset supporting the conclusions of this article is included within the article.
